# Obituary

**Published:** 1886-01

**Authors:** 


### Obituary.

Died in Hartford, Conn., November 11th, Dr. John M. Riggs, D.D.S., aged 75 years, after an illness of about two weeks.

Dr. Riggs was for more than 40 years a dental surgeon in Hartford; he was born in Seymour, Conn., October 25th, 1810. He graduated at Trinity College, in Hartford, in 1837; soon after commenced his studies in dental surgery and has practiced since 1840. He achieved marked distinction in his profession, and in some features of its practice he stood at its very head. He was awarded special honors by dental colleges in Baltimore in 1854 and in 1879, and has been a lecturer there since, both clinical and didactic, and has also given clinical lectures at Harvard University, and has been an occasional contributor to dental surgical periodicals. Dr. Riggs was second only to Dr. Horace Wells in the discovery of anæsthesia, and took in that work a part scarcely less dangerous than Dr. Wells. The latter risked his life; the former risked the being party to Wells' death, had it occurred. Dr. Riggs achieved a wide reputation and high honor in his profession in another direction. Nearly 40 years ago he originated a method, entirely surgical, of treatment of the disease known as pyorrhœa alveolaris, and his treatment attracted such attention that his name was given to it, and for years the disease has been called "Riggs' Disease." No other professor had such success in the treatment of this disease as he; patients came to him from distant places for treatment. His first patient treated for this trouble was a Mr. Wells, as early as 1845, and it was not until sometime afterward, when fortified by experience, he made known his success to the profession. During later years his advice and instruction have been sought by many in the profession. He was a man of marked individuality of character; an independent thinker. He was a man fearless in expressing his views on all matters, and he always had an opinion. He was a student in his profession, and in various directions in science, he kept abreast with the progress of the time in many fields; he was a man of strong mind and sterling integrity. His name will ever have a place in the history of his chosen profession for that which he added to its resources and made so available to others. His life was one of usefulness and his memory will ever be revered by all who knew him.

---

Died in Louisville, Ky., November 11th, Dr. W. G. Redman, aged 66 years.

Dr. Redman has been in ill health for three or four years, and for several months past was quite feeble, though no immediate danger was anticipated. He talked cheerfully and pleasantly with his family and physician in the evening, retiring as usual, but about 12 o'clock arose for water and he seemed then to be suffering quite severe pain in the region of the heart. He had gone but a few steps when he fell senseless to the floor, and was dead in a few minutes. Dr. Redman was born near Eldridge,

Onondago Co., N. Y., April 2, 1821. His father was a prominent physician there, and the son received a good education and studied medicine. The results of this early training were shown throughout his professional life. In 1843 he came to Shelby Co., in Kentucky, where he taught school, and subsequently practiced medicine until 1849 when he married Miss Mary C. Chisem, of Lexington, and removed to Henderson. In 1860 he settled in Louisville and began the practice of dentistry, which he had also studied. He was a fine operator and soon had an extensive and lucrative practice. He was one of the founders of the Kentucky State Dental Association, as also of the Southern Dental Association; he was president of both of these bodies. He continued the practice of his profession until a few months before his death. His failing health compelled him to relinquish practice. Dr. Redman was affable and agreeable, was a favorite with all who came in contact with him; a man of honor and sterling integrity. He leaves a wife and ten children, most of the latter have attained adult life and are occupying useful and honorable positions.

———

Dr. J. H. Bedford died at his home, No. 116 East Chestnut street, Louisville, Ky., at 8 o'clock, Nov. 11th. He had been seriously ill with kidney trouble for several weeks.

Dr. Bedford was born in Bourbon County, August 16th, 1838, where he was raised and educated. He studied dentistry early in life, and removed to this city about twenty years ago to practice his profession. He built up a large practice and was recognized as one of the best dentists in the city. He had an extensive acquaintance both here and throughout the State.

